# Supplementary material for: Transcription Factor Networks in Leaves of Cichorium endivia: New Insights into the Relationship between Photosynthesis and Leaf Development
Source: Plants (Basel). 2019 Nov 21;8(12):531. doi: 10.3390/plants8120531 (PMC6963412; doi:10.3390/plants8120531)
Supplement: Supplementary file 1 [file plants-08-00531-s001.zip › plants-635826-supplementary/Cartella_submission/Figure S1.pdf]

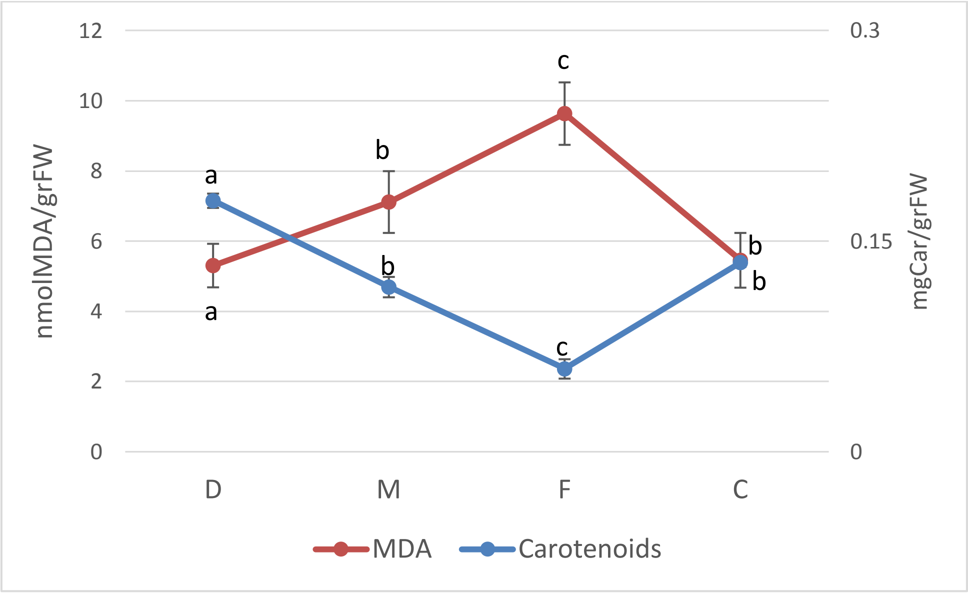

Figure S1. Ratio of leaf dry mass to leaf area (LMA) and ratio of leaf length to leaf width (LI) in 'Domari', 'Myrna', 'Flester' and 'Confiance' plants (Mean ± SD, n = 6).
