# Supplementary material for: Transcription Factor Networks in Leaves of Cichorium endivia: New Insights into the Relationship between Photosynthesis and Leaf Development
Source: Plants (Basel). 2019 Nov 21;8(12):531. doi: 10.3390/plants8120531 (PMC6963412; doi:10.3390/plants8120531)

Figure S1. Determination of the optimum number of clusters for K-means analysis: **(a)** output of the sum of squared error (SSE) method; **(b)** output the average silhouette width method

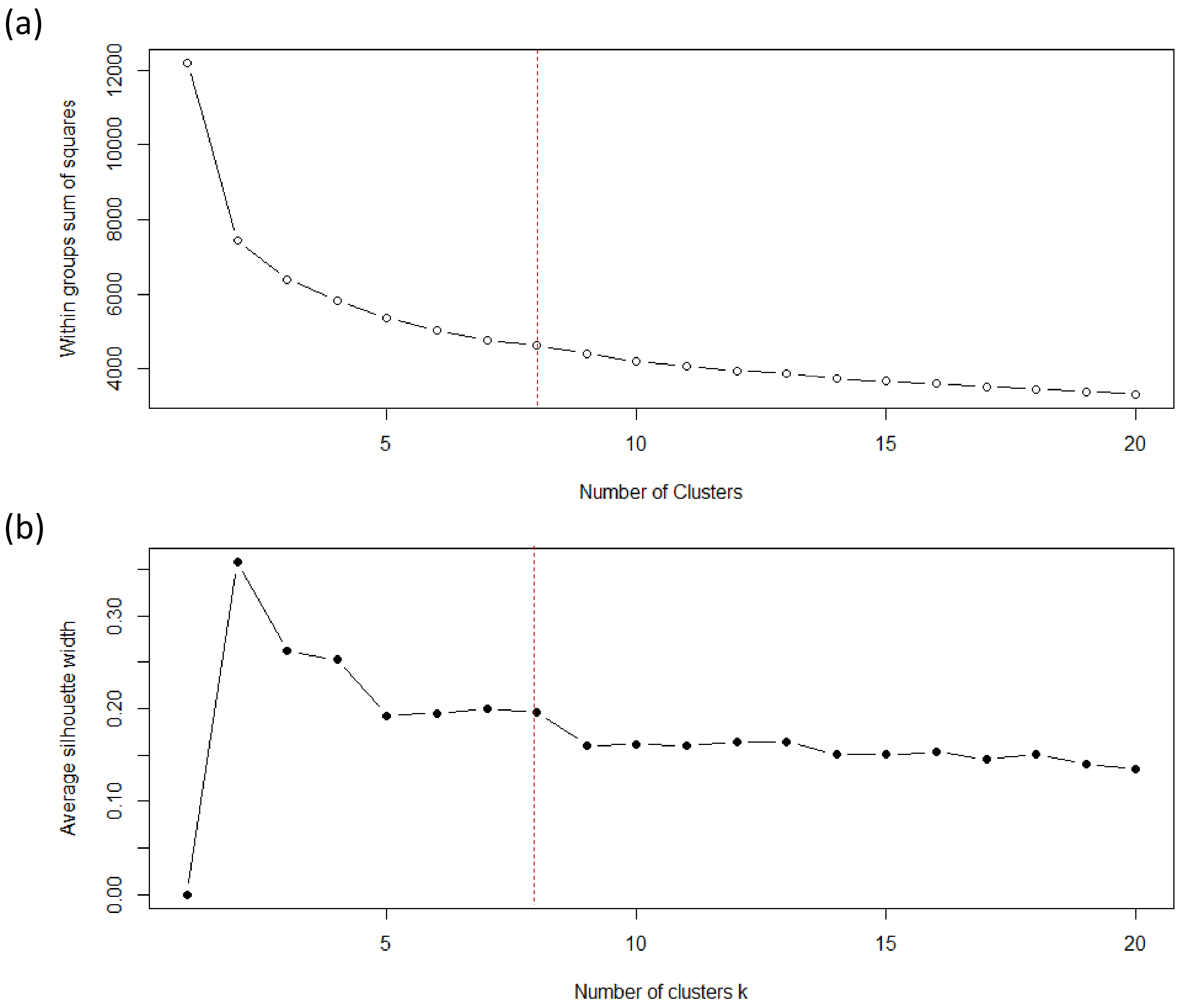

Figure S2. Determination of the optimum number of clusters for K-means analysis: **(a)** output of Calinski–Harabasz index method; **(b)** output of the Gap statistic method

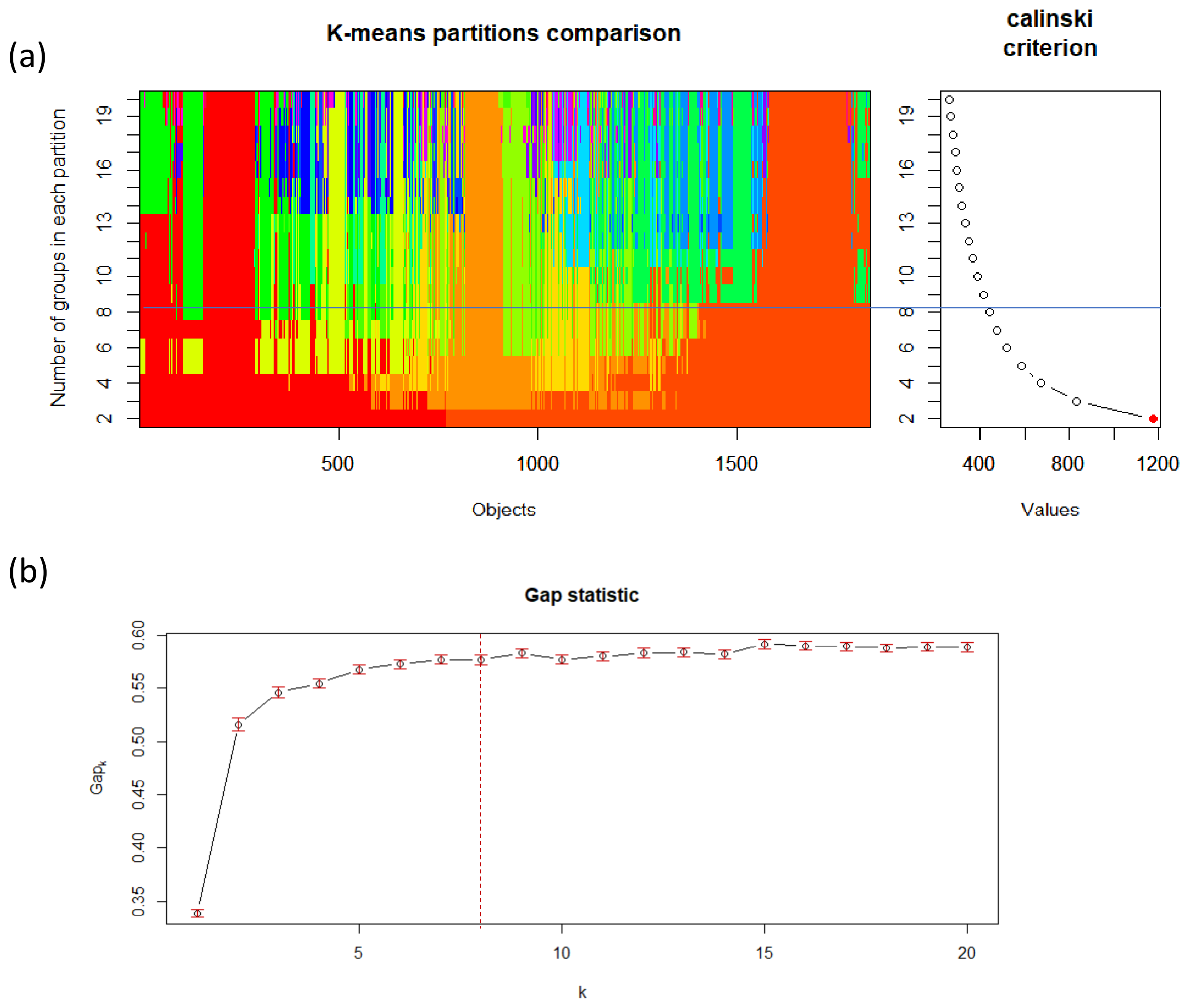

Figure S3. Determination of the optimum number of clusters for K-means analysis: **(a)** heat map of the hierarchical clustering analysis; **(b)** a posteriori validation of cluster number using maximum centroid correlation values with threshold below 0.8

(a)

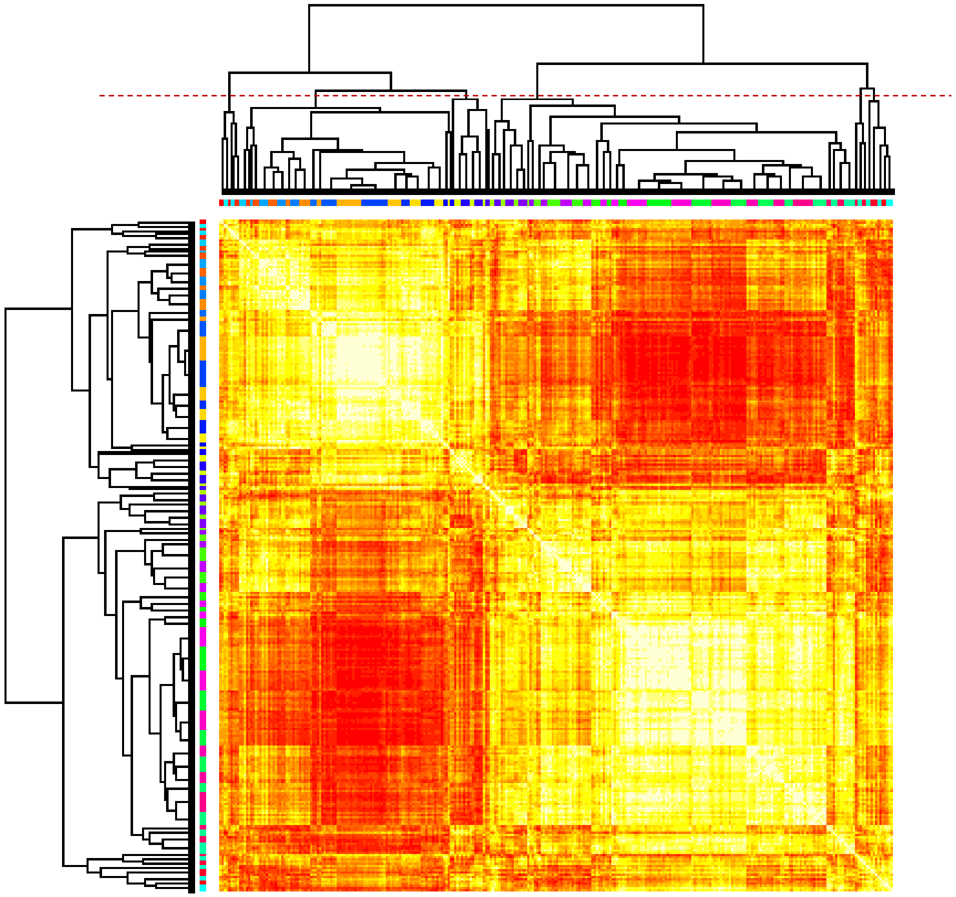

(b)

Cluster	2	3	4	5	6	7	8	9
Max CC	-0.8	0.3	0.4	0.6	0.6	0.7	0.7	0.8

MaxCC = maximum centroid correlation

Figure S4. Correlation of cluster centroids for K-means cluster number = 8 : **(a)** plot of cluster centroids; **(b)** matrix of cluster centroids correlation values

(a)

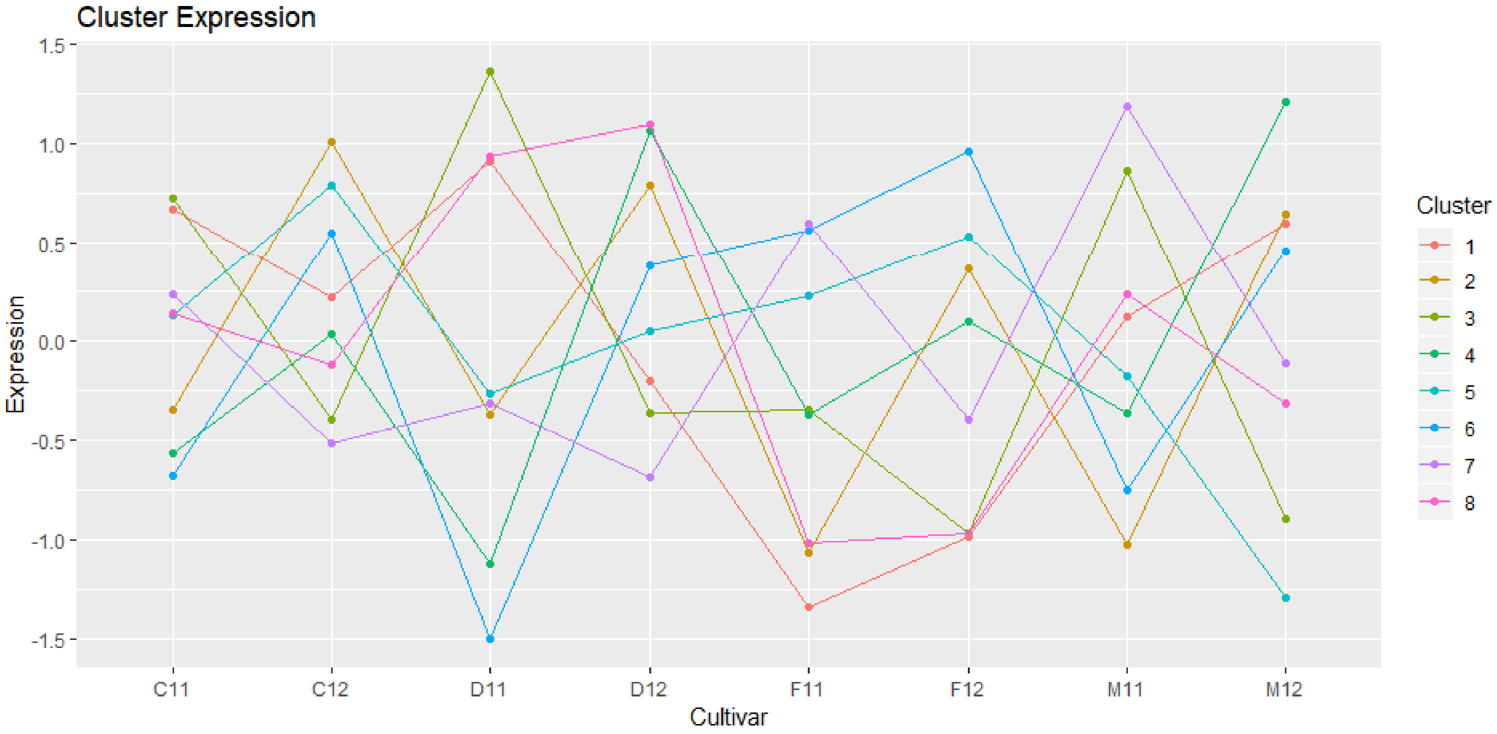

(b)

	1	2	3	4	5	6	7	8
1	1.0000000	0.18024702	0.55554680	-0.11655935	-0.45831518	-0.6816586	-0.1256871	0.65279976
2	0.1802470	1.00000000	-0.58282513	0.67701213	0.05389163	0.5027264	-0.8572353	0.14563030
3	0.5555468	-0.58282513	1.00000000	-0.76288190	-0.05100912	-0.9724148	0.3864120	0.59100680
4	-0.1165593	0.67701213	-0.76288190	1.00000000	-0.35019511	0.6670928	-0.3856859	-0.03170325
5	-0.4583152	0.05389163	-0.05100912	-0.35019511	1.00000000	0.2494987	-0.1664034	-0.19244506
6	-0.6816586	0.50272644	-0.97241478	0.66709285	0.24949874	1.0000000	-0.3173104	-0.63428386
7	-0.1256871	-0.85723529	0.38641205	-0.38568589	-0.16640337	-0.3173104	1.0000000	-0.25311154
8	0.6527998	0.14563030	0.59100680	-0.03170325	-0.19244506	-0.6342839	-0.2531115	1.00000000

Figure S5. Ratio of Malondialdeyde (MDA) content and Carotenoids measurement in 'Domari', 'Myrna', 'Flester' and 'Confiance' plants. (Mean ± SD, n = 3)

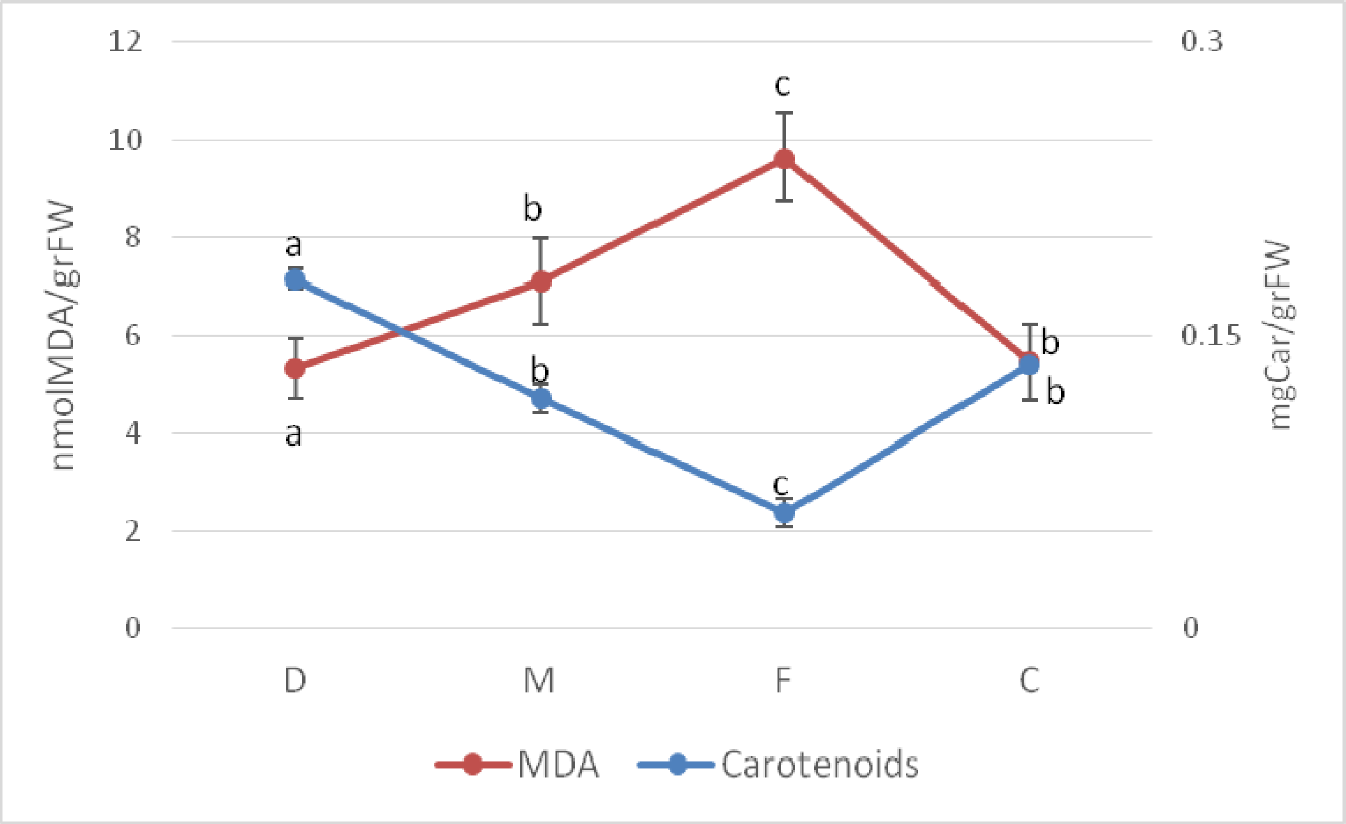

Supplement: Supplementary file 1 [file plants-08-00531-s001.zip › plants-635826-supplementary/Cartella_submission/Supplemental Figures S1-S5.pdf]
