# Supplementary material for: Transcription Factor Networks in Leaves of Cichorium endivia: New Insights into the Relationship between Photosynthesis and Leaf Development
Source: Plants (Basel). 2019 Nov 21;8(12):531. doi: 10.3390/plants8120531 (PMC6963412; doi:10.3390/plants8120531)
Supplement: Supplementary file 1 [file plants-08-00531-s001.zip › plants-635826-supplementary/Cartella_submission/Table S5.pdf]

Table S5. Chlorophyll fluorescence parameters (maximal quantum efficiency (F_v/F_m) measured in dark adapted leaves and quantum efficiency of PSII photochemistry (Φ PSII) measured at steady state with light intensity of 370 µmol photons m^{-2} s^{-1}) were obtained from 6 fully developed leaves of endives (cv. 'Domari' and 'Myrna') and escaroles (cv. 'Flester' and 'Confiance') cultivars grown under greenhouse conditions for X days (average data ± SE; n=6). Four AOI (Area Of Interest) were selected, two in the internal part of the leaf and two in the external part in order to evaluate spatial heterogeneity. For each parameter a one-way analysis of variance (ANOVA) was applied and data followed by common letters in the same row are not significantly different (Student-Newman-Keuls test, $P \leq 0.05$).

Cultivar	F_v/F_m (rel. un.)			Φ PSII (rel. un.)		
	external	internal	*P*	external	internal	*P*
Domari	0.792 ± 0.001 a	0.794 ± 0.001 a	0.32	0.410 ± 0.004 a	0.401 ± 0.006 a	0.23
Myrna	0.807 ± 0.001 a	0.806 ± 0.001 a	0.58	0.379 ± 0.005 a	0.371 ± 0.005 a	0.29
Flester	0.828 ± 0.001 a	0.827 ± 0.001 a	0.45	0.379 ± 0.007 a	0.371 ± 0.003 a	0.30
Confiance	0.780 ± 0.003 a	0.782 ± 0.001 a	0.72	0.423 ± 0.009 a	0.416 ± 0.005 a	0.56
