# Supplementary material for: Transcription Factor Networks in Leaves of Cichorium endivia: New Insights into the Relationship between Photosynthesis and Leaf Development
Source: Plants (Basel). 2019 Nov 21;8(12):531. doi: 10.3390/plants8120531 (PMC6963412; doi:10.3390/plants8120531)
Supplement: Supplementary file 1 [file plants-08-00531-s001.zip › plants-635826-supplementary/Cartella_submission/Table S7.pdf]

Table S7. Several spectral reflectance indices

**Spectral reflectance indices**	**Computations**[1]
Normalized Difference Vegetation Index (NDVI)	$(R_{800} – R_{680}) / (R_{800} + R_{680})$
Photochemical Reflectance Index (PRI)	$(R_{531}-R_{570}) / (R_{531}+R_{570})$
Stress Index (SI)	R_{710} / R_{810}
Water Index (WI)	R_{900} / R_{970}
Structural Independent Pigment Index (SIPI)	$(R_{800} – R_{445}) / (R_{800} + R_{680})$
Normalized Phaeophytinization Index (NPQI)	$(R_{415} – R_{435}) / (R_{415} + R_{435})$

1, R indicates the reflectance in a band centered at a specific wavelength λ
