# Supplementary material for: Transcription Factor Networks in Leaves of Cichorium endivia: New Insights into the Relationship between Photosynthesis and Leaf Development
Source: Plants (Basel). 2019 Nov 21;8(12):531. doi: 10.3390/plants8120531 (PMC6963412; doi:10.3390/plants8120531)
Supplement: Supplementary file 1 [file plants-08-00531-s001.zip › plants-635826-supplementary/Cartella_submission/TableS6.pdf]

Table S6. Primer list.

Gene ID	Amplicon (bp)	Name	Tm	Sequence (5′-3′)
Ce_contig84656	172	BRX_1-F	62.9	GCCTTGCTCCGGATCGTCAA
		BRX_1-R	63.2	TCCACCACTCGAAAGCCCCT
Ce_contig50460	200	ERF_72-F	64.1	GCTGACCGCCGACCTTTTACG
		ERF_72-R	64.4	ACCGGAGCAGAGCCTTTGGAG
Ce_contig5865	83	MYB_77-F	62.6	ATGTTGAGGAAAGGTGCATGGACT
		MYB_77-R	63.1	GTGCCACTTCCCTTCGCCAT
Ce_contig75081	135	Lhcb6_1-F	62.2	CATCCTTCTTGTCCGGCGGA
		Lhcb6_1-R	63.0	CGGCGGGGATCCAGGATTTC
Ce_contig85637	194	SIG_5-F	62.3	GCTTTCCAATTGCCGAATCCGT
		SIG_5-R	62.0	TCGAAAGCCCGGACACAAGT
Ce_contig68433	117	ARR-B_7-F	62.7	CTGGCATGCTTGGTAGGCTCA
		ARR-B_7-R	62.3	TTGCGGGTACAACTGGCTGT
Ce_contig1428	121	G2-like_1-F	63.0	CCACCACCAGCACCACATGT
		G2-like_1-R	63.2	TGGCATGGGGTGGTGGGATA
Ce_contig68890	75	ACT-F	61.9	GCCAAATCCAGCTCATCAGTCG
		ACT-R	61.6	TCTTTCGGCTCCGATGGTGAT
